# Tongue Base Ectopic Thyroid Tissue—Is It a Rare Encounter?

**DOI:** 10.3390/medicina59020313

**Published:** 2023-02-08

**Authors:** Balica Nicolae Constantin, Trandafir Cornelia Marina, Stefanescu Horatiu Eugen, Enatescu Ileana, Gluhovschi Adrian

**Affiliations:** 1 ENT Department, Victor Babeş University of Medicine and Pharmacy, Bd. Revolutiei No. 6, 300054 Timisoara, Romania; 2 Victor Babeş University of Medicine and Pharmacy Timisoara, Piaţa Eftimie Murgu Nr. 2, 300041 Timisoara, Romania; 3 NeoNatology Department, University of Medicine and Pharmacy Timisoara, 300041 Timisoara, Romania; 4 Gynecology Department, University of Medicine and Pharmacy Timisoara, 300041 Timisoara, Romania

**Keywords:** tongue base, ectopic thyroid tissue, dysphagia, upper airway obstruction

## Abstract

Failure in the embryological development of the thyroid in adults is rarely seen. We present the case of a 79-year-old female patient who complained of dysphagia and progressive upper respiratory obstruction, which started 12 months prior to her admission. An ENT clinical exam revealed a tongue base, spherical, well-defined tumour covered by normal mucosa. Further assessments established the diagnosis of the tongue base ectopic thyroid tissue. Due to the patient’s symptoms, a transhyoid tongue base tumour removal was performed. The selected patient gave consent for participation and inclusion in this paper, in compliance with the 1964 Helsinki declaration.

## 1. Introduction

Ectopic thyroid tissue is an embryologic defect. The disease involves the aberrant development of the thyroid gland from the foramen caecum to its inferior cervical position [1,2]. The causes and exact mechanism of this aberrant defect have not been clearly elucidated, even though some transcription factors seem to playa role in the migration of the thyroid [3]. Its prevalence is about 1 per 100,000–300,000 people, increasing to 1 per 4000–8000 in patients with thyroid disease [1,2]. It is mostly diagnosed in childhood, adolescence, and around menopause, with a slight prevalence in females [4].

Ectopic thyroid tissue can be found anywhere along the course of the thyroglossal duct [5]. The base of the tongue represents the most frequent location [6]. Other sites involved are the anterior tongue, the submandibular or sublingual region, the larynx, the trachea, the mediastinum, and the heart [7,8]. The differential diagnosis of ectopic thyroid tissue must include metastatic thyroid carcinoma [9].

Most ectopic thyroids are asymptomatic. The symptoms are related to the sites of the ectopic tissue and can cause dysphagia, dysphonia, bleeding, or even upper respiratory obstruction, and, therefore, patients are referred to an ENT specialist for their diagnosis and treatment.

The combination of diagnostic imaging techniques and hormonal biological examinations correlating with the clinical ENT examination plays a fundamental role in the diagnosis of an ectopic thyroid tissue.

There is no consensus for the optimal treatment of ectopic thyroid tissue due its rarity. Some authors recommend a “wait-and-see” policy to ascertain whether the patient is in a euthyroid status or if the asymptomatic ectopic tumour is small. If the tumour continues to enlarge, leading to the compression of surrounding structures with consecutive occurrences of algia, dysphagia, dysphonia, or upper respiratory obstruction, surgical treatment can be proposed. Conventionally, various external approaches have been described in the literature; recently, transoral surgery has gained increasing popularity as an alternative to external surgical treatment. Suppressive hormone therapy and ablative radioiodine therapy are described as alternative treatments for patients with lingual thyroid by some authors [3,4,10].

It is important to establish the periodic follow-up of patients with an endocrinologist specialist due to the post-operative hypothyroidism risk.

## 2. Case Report

A 79-year-old female patient with dysphagia and respiratory insufficiency was admitted to the ENT department. The symptomatology started twelve months prior to her admission to the hospital. She had no history of smoking and no alcohol intake. She was under medical treatment for hypercholesterolemia and high blood pressure.

**Figure 1:**
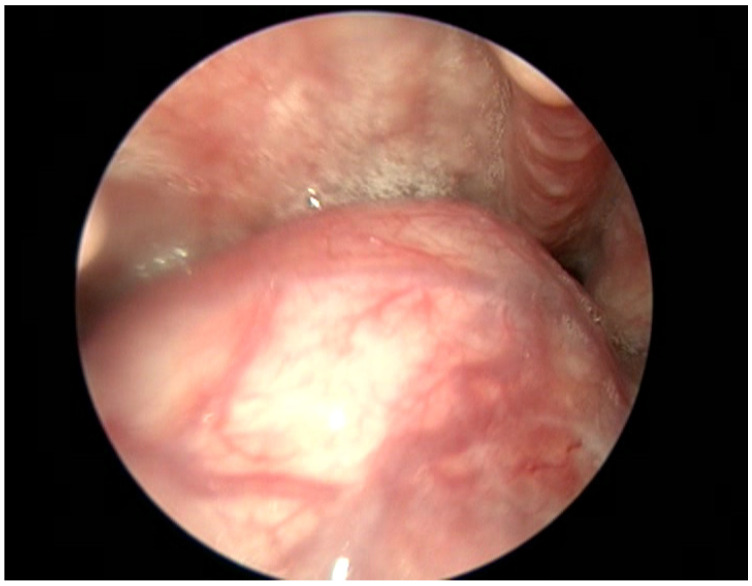
70 degrees rigid hypopharyngoscope shows the mass in the sublingual region.

The clinical ENT examination revealed no cervical adenopathy. The endoscopic examination (with a 70 degree rigid hypopharyngoscope) revealed a tongue base, spherical, solid, well-defined tumour covered by normal mucosa and obstructing the visualisation of the larynx (Figure 1).

**Figure 2:**
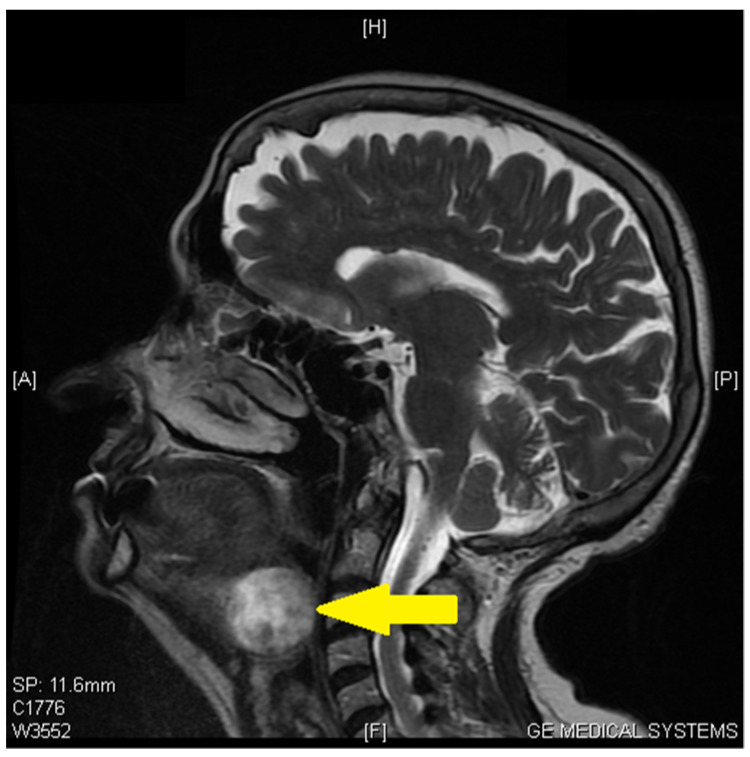
Magnetic Resonance Imaging (MRI) imaging shows a mass in the sublingual region (arrow).

By comparing the further assessments (neck ultrasonography, thyroid scintigraphy, and cervical region Magnetic Resonance Imaging MRI (Figure 2)), the diagnosis of a tongue base ectopic thyroid tissue was established.

**Figure 3:**
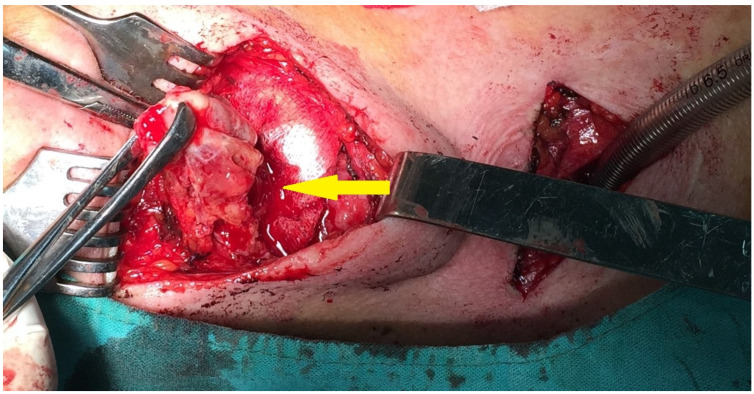
Intraoperatory imaging (Arrow—Tumor).

Due to the patient’s symptomatology, the decision was made to perform surgical treatment with an external approach. Under general anaesthesia, we performed a tracheotomy and a transversal superior incision at the level of the hyoid bone, externally excising the tongue base tumour (Figure 3).

The tracheotomy was performed for airway control following surgery. Nutrition was delivered via a naso-gastric feeding tube. The post-operative treatment included a large spectrum of antibiotics for prophylaxis, anti-reflux medical treatment, and non-opioid analgesics. The post-operative evolution was unremarkable. The tracheotomy cannula and the feeding tube were both maintained for seven days, and deglutition was normal after the removal of the feeding tube. The patient was free of symptoms two weeks after the surgical procedure. The patient was referred to an endocrinologist before and after the surgery for hormonal substitutive treatment. Circulating levels of thyroid hormones at baseline were thyroid stimulating hormone TSH 12.8 μU/mL, FT3 1.9 pg/mL, and FT4 8.9 pg/mL.

The patient started L-T4 replacement therapy 5–7 days after the surgery. The value of L-T4 was 100 μg per day. After two month under L-T4 replacement therapy, the TSH was3.0 μU/mL; FT 3 was 4.1 pg/mL;and FT4 was 11.5 pg/mL.

The anatomo-pathological exam revealed the ectopic thyroid tissue.

Six years after the surgery, there was no evidence of recurrence and no symptomatology.

## 3. Discussion

The first endocrine gland that occurs during foetal development is the thyroid gland [11].

The embryologic development implies an endodermal diverticulum (third or fourth week of gestation), which descends from the foramen caecum to the thyroid gland’s final location (24th day) through the thyroglossal duct. The latter undergoes atrophy prior to the definitive formation of the thyroid [1,12,13]. Failure in thyroid migration along the path from the original region of the thyroid to its final cervical location causes ectopic thyroid tissue, which can be commonly found at any location from the tongue base to the mediastinum [14,15].

Many causes have been linked to the failure of thyroid migration, as well as to atypical morphological aspects of the thyroid, notably molecular, genetic, or epigenetic disorders [16,17].

In 70–90% of cases of ectopic foci of thyroid tissue, the ectopic tissue is the only thyroid tissue present, and the thyroid gland is absent [3,6,18]. It may occur at any age, from 5 months to 40 years, being more common at younger ages, with a high prevalence in females [1,2,19,20]. In the literature, only a few cases of a dual ectopic thyroid gland along with a cervical thyroid gland have been described [21]. In the literature, half of patients are euthyroid, and the rest are hypothyroid [3,6]. In our case the patient was referred to an endocrinologist for the supplementation hormonal treatment and follow-up.

The presence of an ectopic thyroid tissue can be asymptomatic, can cause local symptoms such as dysphagia, and can be the leading cause of death as described by Dr Hickman in a case report of a new-born with a lingual thyroid who died from severe respiratory distress [7]. All diseases that can affect a normal thyroid can affect the ectopic thyroid tissue (adenoma, hyperplasia, inflammation, and rarely malignancy) [6,22]. The incidence of malignancy is estimated at 1% [19,23,24]. 

Massine et al. described a case of a 57-year-old patient with the diagnosis of a papillary carcinoma arising from a lingual thyroid and an invasive keratinising poorly differentiated squamous cell carcinoma from adjacent structures. The patient was treated by a total glossectomy, with a left elective neck dissection, supraglottic laryngectomy, and midline mandibulotomy with a rectus free flap [24].

The presence of an ectopic thyroid tissue is less frequently encountered in other anatomical spaces: the pancreas [25], porta hepatis [26], submucosa of the duodenum [27], and the iris [28]. In these cases, it is important for the clinician to rule out metastases from a thyroid carcinoma [29].

Sometimes the thyroid malignancy is excluded, but the ectopic tissue is confirmed at a distance from its normal path of embryological development. In those cases, the congenital defect is difficult to explain. Many theories are present in the literature. For example, the common origin of the intra-abdominal organs and the thyroid are from the endodermal germ layer. The presence of an ectopic thyroid tissue was found by Aiwen Ma et al. below the diaphragm, in a retroperitoneal mass between the superior border of the kidney, having a close interaction with the pancreatic hook [23,30].

However, the theory described previously does not explain the presence of the ectopic tissue in the adrenal gland because the adrenal cortex originates from the mesodermal layer while the medulla originates from the ectodermal layer. Many other explanations can be found in the literature: teratoma, metaplasia, and choristoma [23,31].

When a basilingual mass is found, a complete Ear, nose and throat examination (ENT) exam should be performed, and uncommon diseases should be kept in mind when examining any patient [32]. A cervical ultrasound is a valuable tool for identifying an ectopic thyroid tissue. Computed Tomography (CT) scans and MRI imaging are widely used to assess the exact location and extension of ectopic thyroid foci. Scintigraphy is highly sensitive in detecting thyroid tissue, which is used for the differentiation of the thyroid from other differential diagnoses. As has been previously described in the literature, a biochemical thyroid profile may be necessary in adult patients and in new-borns, as some studies suggest that hypothyroidism can be diagnosed in patients with ectopic thyroid tissue due to the absence of a functioning thyroid gland [11].

Differential diagnoses should include thyroglossal duct cysts without thyroid tissue, midline branchial cysts, lymphangioma, haemangioma, and minor salivary gland tumours [2,33].

If the diagnosis of an ectopic lingual thyroid is established and the patient presents obstructive symptomatology or a malignant disease is suspected, surgical treatment must be performed after a multidisciplinary examination of the patient.

The patients with an airway obstruction may include a tracheostomy as an emergency procedure or for airway control following surgery, as described in our report (severe post-operative oedema is frequent). Even though a tracheostomy is considered a routine surgical procedure, there area number of potential vessels that can be accidentally injured during the procedure. Injuries of the thyroidea-ima were described in some cases of percutaneous tracheostomy [34,35].A study by Laphatrada Yurasakpong et al. revealed the presence of the thyroidea-ima in approximately 3.8% population, with a decline in the prevalence of the thyroidea-ima over the generations. Awareness of this normal variant of neck anatomy is important when performing a tracheostomy [36]. In our report, this variant was not present.

The treatment of an ectopic lingual thyroid depends on several factors. In cases with no clinical symptoms, a substitutive therapy with thyroid hormones can be proposed. Ablative radioiodine therapy is reserved for cases when surgical therapy is contraindicated, and it is not usually used for young patients [13,24]. However, in our report, the size of the lesion, the presence of local symptoms, and the presence of complications made us perform the surgical therapy.

The excision of q lingual ectopic thyroid tissue may be performed with a variety of approaches from intensive surgical procedures (Sistrunk technique, lateral pharyngotomy, and trahnsyoid incision) to simpler and more cost-effective procedures [24,37]. Extensive surgeries offer good results, but complications such as fistula formation, infection, and the presence of a cervical/facial scar may be unsatisfactory for patients with higher cosmetic requirements.

The intraoperatory risk of bleeding during a tongue base surgery is high. However, external surgeries offer better visualisation, especially for large masses, and the bleedings are easy to control during an open surgery. Some authors use midline mandibulotomy and tongue splitting techniques to achieve a good exposure and minimise the risk of injuries [38,39].

Initially used to excise all thyroglossal tract tissue involved in thyroglossal cyst, the Sistrunk technique can be used for the management of an ectopic lingual tissue. It can offer a satisfactory removal, and the majority of the post-operative course is surgically uneventful [40,41].

In our days there has been a progressive trend to approach thyroid ectopic tissue using transoral surgery/laser methods. A Korean team demonstrated the feasibility of a Sistrunk procedure by robot-assisted surgery via a retroarticular incision for a thyroglossal cyst [42].

Trans-oral robotic surgery (TORS) is known as a minimally invasive surgical technique, approved in 2009 by the Food and Drug Administration (FDA) for head and neck surgery [43]. It improves access and visualisation, avoiding the classical invasive open techniques (midline labiotomy, tongue splitting, and mandibulotomy) [44,45,46]. Compared to traditional surgeries techniques, it has a different anatomic perspective. It understands the muscular landmarks and vascular anatomy and it is important for minimising the surgical risks, notably the bleeding one. The development of TORS allows for less morbidity and offers better cosmesis post-operatively [47].

In order to avoid substitutive lifelong hormone therapy, in the literature a transplantation of the lingual thyroid tissue in the muscles of the neck has been described [48].

Clearly, there remain certain situations in which minimally invasive methods are not possible. Patient factors and the availability of surgical skills and technologies should be taken into account when managing a patient with a thyroid ectopic tissue.

In our ENT department, there are more than 2500 patients admitted annually. The incidence of an ectopic thyroid among the admitted patients is roughly one case every five years.

As with many previous case reports, we acknowledge that the present article has some limitations. The rarity of this entity in our centre is likely subject to variations in clinical practice outcomes, linked to the hospital turnover of patients and the resources available.

## 4. Conclusions

Ectopic thyroid tissue is a rare developmental anomaly. An accurate ENT evaluation and differential diagnoses should increase the suspicion of an ectopic thyroid tissue. In cases with mild symptoms, substitutive hormone treatment might be implied, while in cases with deglutition or respiratory impairment, surgical treatment seems to be the best option.
